# Influence of Molecular Weight and Temperature on the Pyrolysis Behavior of Polyethylene

**DOI:** 10.3390/polym17050576

**Published:** 2025-02-21

**Authors:** Eunji Chae, Sung-Seen Choi

**Affiliations:** Department of Chemistry, Sejong University, 209 Neungdong-ro, Gwangjin-gu, Seoul 05006, Republic of Korea; eunji.chae@sejong.ac.kr

**Keywords:** polyethylene, pyrolysis temperature, molecular weight, alkanes, alkenes, alkadienes

## Abstract

The pyrolysis technique is a useful recycling method for waste polyethylene (PE). Various PEs with different molecular weights have been produced and are widely used. The major pyrolysis products of PE include alkadienes (C_n_H_2n−2_), alkenes (C_n_H_2n_), and alkanes (C_n_H_2n+2_). In this study, the differences in pyrolysis behavior of PE based on its molecular weight and the pyrolysis temperature (423–764 °C) were investigated using four types of PEs, with M_w_ = 2.0 × 10^3^, 16.0 × 10^3^, 28.3 × 10^3^, and 56.8 × 10^3^ g/mol. More specifically, the pyrolysis products were compared in terms of their type (alkanes, alkenes, and alkadienes) and size (the number of carbon atoms). The order of production was alkenes > alkanes > alkadienes. The alkadiene/alkene ratios tended to increase with the PE molecular weight and size of the pyrolysis products. The alkadiene/alkene ratio increased until the pyrolysis temperature reached 670 °C. The alkane/alkene ratios tended to decrease as the PE molecular weight increased; however, they increased with the increasing size of the pyrolysis products. The alkane/alkene ratio decreased as the pyrolysis temperature increased, until it reached 670 °C. The formation of alkenes was more favorable than that of alkadienes and alkanes.

## 1. Introduction

Polyethylene (PE) can be classified into high-density PE (HDPE), low-density PE (LDPE), linear low-density PE (LLDPE), very-low-density PE (VLDPE), high molar weight HDPE (HMW-HDPE), and ultra-high molar weight PE (UHMWPE) based on its density, molecular weight, and degree of branching [1,2]. PE is a general-purpose thermoplastic that accounts for more than one-third of the global plastic market [1,2]. In 2023, global plastic production reached 413.8 million tons, of which 12.2% was HDPE and 14.0% was LDPE and LLDPE [3].

PE is widely used owing to its low cost, easy processability, excellent electrical insulation and chemical resistance, moderate transparency, lack of odor, and non-toxicity [1,2,4]. It is primarily used as a packaging film in the fields of food, construction, agriculture, and manufacturing goods [1,5]. Moreover, PE is also used in food packaging trays, diapers, absorbent pads, textiles, and fishing nets [5]. HDPE is commonly used in milk bottles, household chemicals, personal toiletries, medicine packaging, and various water and gas pipes [5]. It is also widely used in cables and other wire coverings owing to its excellent electrical insulation properties.

As the consumption of PE products increases, they pose a serious environmental problem worldwide because PE is not easily decomposed and remains in the environment for a long time. Therefore, the method of disposing of this plastic waste remains a critical issue. LDPE and HDPE constitute the second and third largest types of plastic waste in municipal solid waste, respectively [6,7]. In the US, the recycling rates of HDPE and LDPE/LLDPE in 2015 were 10.3% and 6.2%, respectively [8]. Currently, landfilling remains the main method of waste disposal; however, it is becoming undesirable because of legal sanctions, increased costs, and the low biodegradability of polymers [9].

Primary recycling, mechanical recycling, chemical recycling, and energy recovery methods are mostly used for PE recycling [9,10,11,12]. Mechanical recycling involves the separation of polymers from associated contaminants and their reprocessing via melt extrusion [13]. Among the mechanical recycling technologies, dissolution/reprecipitation uses a solvent/nonsolvent system to separate and recycle polymers [9]. In solvent-based processes, plastic waste is treated with a solvent to dissolve the polymer materials and recover them through reprecipitation [9,14,15].

In chemical recycling, long chains are broken down through complete depolymerization into monomers or partial decomposition into other valuable secondary materials [2,13]. Chemical recycling, also known as tertiary recycling, converts waste polymers into their original monomers or other chemicals that can be used as feedstock for various industrial processes or as transportation fuels [13]. Pyrolysis is the most widely used chemical recycling for PE [6,16,17,18]. Pyrolysis at a high temperature of >700 °C produces a C1–C4 olefin mixture and aromatic compounds (benzene, toluene, and xylene), while pyrolysis at a low temperature of 400–500 °C obtains high calorific value gases, condensable hydrocarbon oils, and waxes [13,19,20]. High-temperature pyrolysis of PE maximizes the gas fraction to obtain olefins, which can be used as monomers for reproducing polyolefins [13,21]. In 2022, 16.7% of the plastic waste generated in the UK was landfilled, 55.3% was used for energy recovery, and 28.0% was recycled [13,22].

During the pyrolysis of PE, a mixture of hydrocarbons is formed, the relative proportions of which depend on the process conditions, such as the type of plastic, temperature, pressure, and heating rate [17,23,24]. This pyrolysis occurs via a random fission mechanism that produces a heterogeneous mixture of n-paraffins (alkanes), α-olefins (1-alkenes), and α,ω-dienes (α,ω-alkadienes) over a wide molecular weight range [17,18,25,26,27]. The molecular weight of PE and the pyrolysis temperature can influence the relative abundances of the major pyrolysis products of PE. However, differences in the pyrolysis behaviors of PE, depending on its molecular weight and the pyrolysis temperature, have not been examined in detail. Therefore, this study investigated the influence of the molecular weight of PE and the pyrolysis temperature on the production of major pyrolysis products using four PEs with different molecular weights. Five pyrolysis temperatures of 423, 500, 590, 670, and 764 °C were employed. The pyrolysis products were compared in terms of their type (alkanes, alkenes, and alkadienes) and size (the number of carbon atoms). Energies of some neutral species, radicals, and biradicals based on a model linear saturated hydrocarbon, C_30_H_62_, were calculated to compare favorability for the formation of major pyrolysis products of PE.

## 2. Materials and Methods

### 2.1. Materials

Four polyethylene (PE) samples with different molecular weights, namely PE2k, PE16k, PE28k, and PE56k, were purchased from Polymer Standard Service GmbH (Mainz, Germany). Their number-average molecular weights (M_n_) were 1.7 × 10^3^, 10.7 × 10^3^, 21.0 × 10^3^, and 42.3 × 10^3^ Da, respectively, while their weight-average molecular weights (M_w_) were 2.0 × 10^3^, 16.0 × 10^3^, 28.3 × 10^3^, and 56.8 × 10^3^ Da, respectively.

### 2.2. Pyrolysis–Gas Chromatography/Mass Spectrometry (Py-GC/MS)

The sample weight for Py-GC/MS was approximately 0.2 mg, and each sample was analyzed 4 times. Py-GC/MS analysis was carried out using an Agilent 6890 gas chromatograph equipped with a 5973 mass spectrometer (Agilent Technology Inc., SantaClara, CA, USA) and a JCI-55 Curie point pyrolyzer (Japan Analytical Industry Co., Tokyo, Japan). Pyrofoils with Curie points of 423, 500, 590, 670, and 764 °C were used. Pyrolysis was performed for 10 s using the pyrofoil under a helium (He) atmosphere. A DB-5MS capillary column (30 m × 0.32 mm, 25 μm film thickness) (Agilent Technology Inc., USA) was used. The injector temperature was 250 °C. The inlet temperature was 250 °C, the split ratio was 1:15, and helium (1.8 mL min^−1^) was used as the carrier gas. The GC oven temperature program was as follows: 30 °C (held for 3 min) to 50 °C (held for 3 min) at a rate of 10 °C min^−1^, to 180 °C (held for 1 min) at a rate of 10 °C min^−1^, and then to 250 °C (held for 3 min) at a rate of 10 °C min^−1^. The GC/MS interface temperature was 250 °C. The electron ionization (70 eV) was used to ionize the pyrolysis products. The MS source temperature was 230 °C.

### 2.3. Calculations

We obtained energy-minimized structures of the neutral, radical, and biradical species formed from PE with a series of electronic structure calculations. A series of electronic structure calculations were carried out to obtain structures and energies of the target species. Base structures were created using Avogadro software. We performed simulated annealing with Spartan’10 [28] to find low energy structures using a molecular mechanics force field (MMFF94) [29,30], as implemented in Spartan’10. From this, low energy conformers were obtained. All DFT calculations were performed with a B3LYP/6-31++G(d,p) level using the energy and frequency optimization option by Gaussian 09 [31]. From the DFT calculations, the lowest energy structure was obtained as the final, best structure.

## 3. Results and Discussion

### 3.1. Principal Pyrolysis Products Formed from PE

**Figure 1:**
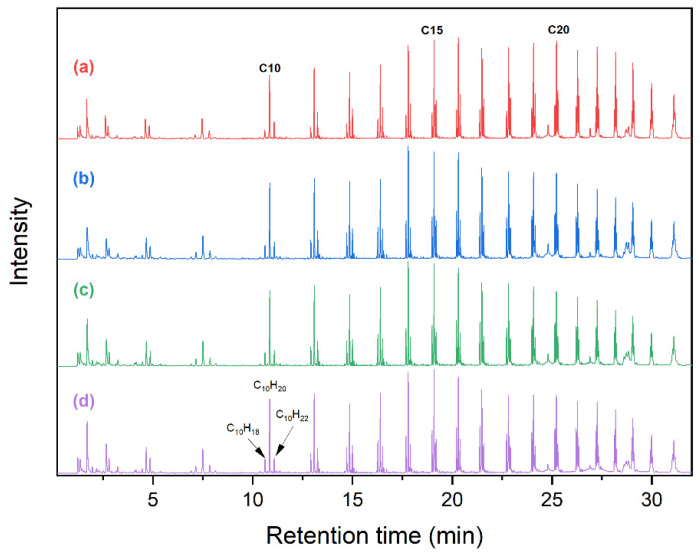
Py-GC/MS chromatograms at 590 °C for PE2k (**a**), PE16k (**b**), PE28k (**c**), and PE56k (**d**).

Figure 1 illustrates the representative Py-GC/MS chromatograms of the four PE samples at 590 °C. The major pyrolysis products were alkadienes (C_n_H_2n−2_), alkenes (C_n_H_2n_), and alkanes (C_n_H_2n+2_). The relative intensities of the major pyrolysis products varied based on the molecular weight of the PE sample and the size of the pyrolysis product. Alkenes were more abundant than alkadienes and alkanes. The most abundant pyrolysis product was C_n_H_2n_ (n = 14, 15, or 16).

**Scheme 1:**
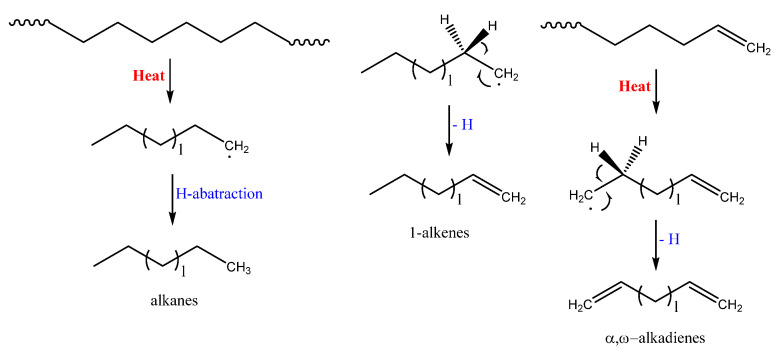
Formation mechanisms of alkanes, 1-alkenes (alkenes), and a,w-alkadienes (alkadienes) from polyethylene by pyrolysis.

The formation of alkanes, alkenes, and alkadienes from PE occurs via the dissociation of carbon–carbon single bonds (C-C) upon heating, and when various radicals of CH_3_(CH_2_)_n_^•^ are generated (Scheme 1). Alkanes are formed when the CH_3_(CH_2_)_n_^•^ radicals take a hydrogen atom from another PE chain. Alkenes are generated by the rearrangement of radicals, which results in the loss of a hydrogen atom. If another radical (the second radical) is formed on the other side of the alkene, an alkadiene can be produced. Alkadienes may also be generated from the biradicals of ^•^(CH_2_)_n_^•^, following rearrangement to lose two hydrogen atoms.

### 3.2. Influence of Pyrolysis Temperature and PE Molecular Weight on Generation of Pyrolysis Products

**Figure 2:**
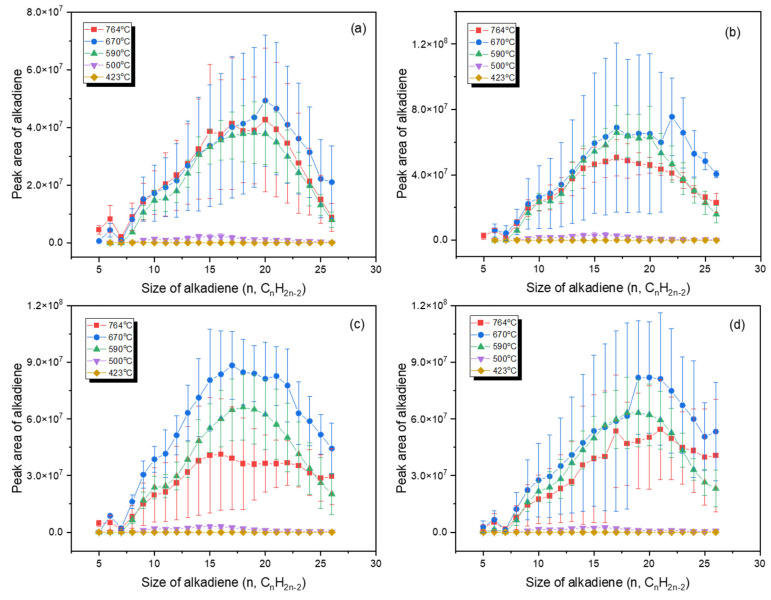
Variations in the peak areas of alkadienes produced by pyrolysis with the size of alkadiene (carbon number) for PE2k (**a**), PE16k (**b**), PE28k (**c**), and PE56k (**d**).

Alkadienes smaller than C_8_H_14_ were produced in trace amounts (Figure 2). The abundance of alkadienes increased as their size increased from C_7_H_12_ to approximately C_20_H_38_, and then decreased. Alkadienes were not generated at 423 °C, and the production of alkadienes at 500 °C was negligible. This implies that the pyrolysis temperature should be higher than 500 °C to produce alkadienes from PE. The abundance of alkadiene increased as the pyrolysis temperature increased from 590 °C to 670 °C, and then decreased by increasing the pyrolysis temperature to 764 °C.

**Figure 3:**
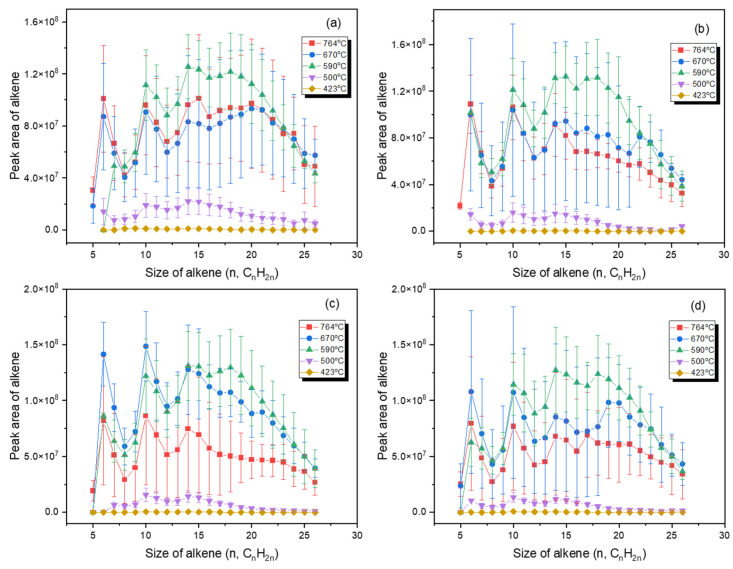
Variations in the peak areas of alkenes produced by pyrolysis with the size of alkene (carbon number) for PE2k (**a**), PE16k (**b**), PE28k (**c**), and PE56k (**d**).

Alkenes were rarely produced at 423 °C: the PE2k sample produced trace amounts of C_8_H_16_–C_21_H_42_, while the other three PE samples generated trace amounts of C_10_H_20_–C_17_H_34_. The variations in the peak areas of alkenes with the pyrolysis product size are depicted in Figure 3. The production of alkenes at 500 °C was not significant. The C_6_H_12_ was not observed at 500 °C for all the samples. The peak areas of alkenes notably increased as the pyrolysis temperature increased from 500 °C to 590 °C, and then decreased following a further increase in the pyrolysis temperature. The peak areas demonstrated a notable increase at the C10 product (C_10_H_20_) and then showed a local minimum for the C12 product (C_12_H_24_). The peak areas decreased after C20 (C_20_H_40_). The abundance of alkenes decreased as the molecular weight of the PE sample increased. The abundances of alkenes generated at 590 °C were greater than those at 670 and 764 °C, and those at 670 °C were larger than those at 764 °C.

**Figure 4:**
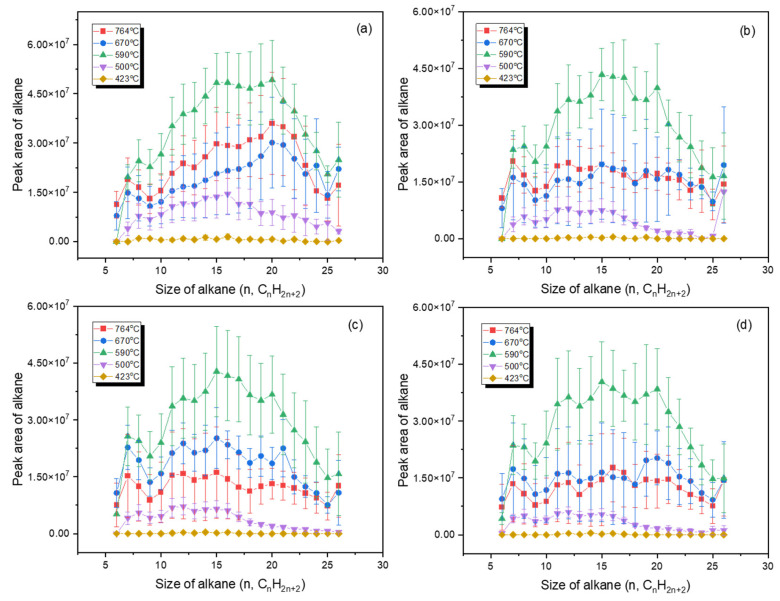
Variations in the peak areas of alkanes produced by pyrolysis with the size of alkane (carbon number) for PE2k (**a**), PE16k (**b**), PE28k (**c**), and PE56k (**d**).

The production of alkanes demonstrated trends similar to those of alkenes (Figure 4). Alkanes were rarely produced at 423 °C: the PE2k sample produced trace amounts of C_8_H_18_-C_21_H_44_, while the other three PE samples generated trace amounts of C_11_H_24_–C_17_H_36_. Compared with the production of alkenes at 500 °C, that of alkanes at 500 °C was increased and the abundances of alkanes tended to decrease slightly as the molecular weight of the PE sample increased. The abundance of alkanes generated at 590 °C was greater than that at 670 and 764 °C, and those at 670 °C were larger than those at 764 °C.

### 3.3. Comparison of Favorability for Productions of Alkanes, Alkenes, and Alkadienes from PE by Pyrolysis

**Figure 5:**
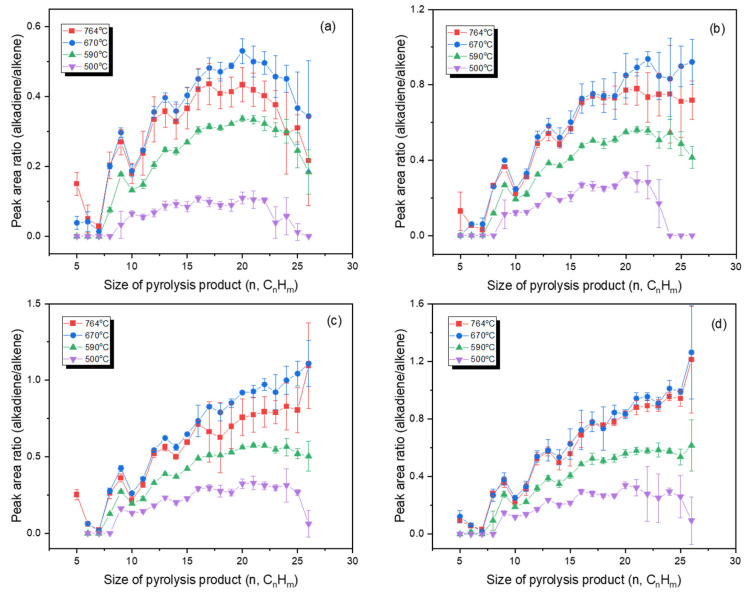
Variations in the peak intensity ratio of alkadiene/alkene with the size of pyrolysis product (carbon number) for PE2k (**a**), PE16k (**b**), PE28k (**c**), and PE56k (**d**).

To compare the production of alkadienes with that of alkenes, their abundance ratios were plotted as a function of the size of the pyrolysis product (Figure 5). The peak intensity ratios of alkadiene/alkene were lower than 1.0, except for few cases (the C25 and C26 species at 670 °C and the C26 one at 764 °C for the PE28k sample, and the C24 and C26 products at 670 °C and the C26 one at 764 °C for the PE56k sample). In particular, the peak intensity ratios of alkadiene/alkene were lower than 0.6 for the PE2k sample. This indicates that the formation of alkenes from PE was more favorable than that of alkadienes. The alkadiene/alkene ratio increased until 670 °C and then slightly decreased as the pyrolysis temperature increased. The ratio increasing with the pyrolysis temperature indicates that the production of alkadienes becomes more favorable at high temperatures. This ratio tended to increase with the molecular weight of the PE sample, indicating that the production of alkadienes is more favorable for PE samples with high molecular weights. The peak intensity ratios showed a local minimum at the C10 product, except for the cases at 500 °C. The peak intensity ratios of the PE2k samples exhibited a maximum for the C20 species. For the higher-molecular-weight samples of PE16k, PE28k, and PE56k, the peak intensity ratios of alkadiene/alkene tended to increase with the size of pyrolysis products, except for the case at 500 °C. This indicates that the formation of a secondary radical to produce an alkadiene is more favorable at a position far from the existing alkene site. For the PE28k and PE56k samples, we found alkadiene/alkene ratios >1.0 in the large pyrolysis products generated at high temperatures of 670 and 764 °C, which means that the production of alkadienes is more favorable than that of alkenes in the case of large pyrolysis products and high pyrolysis temperatures.

**Figure 6:**
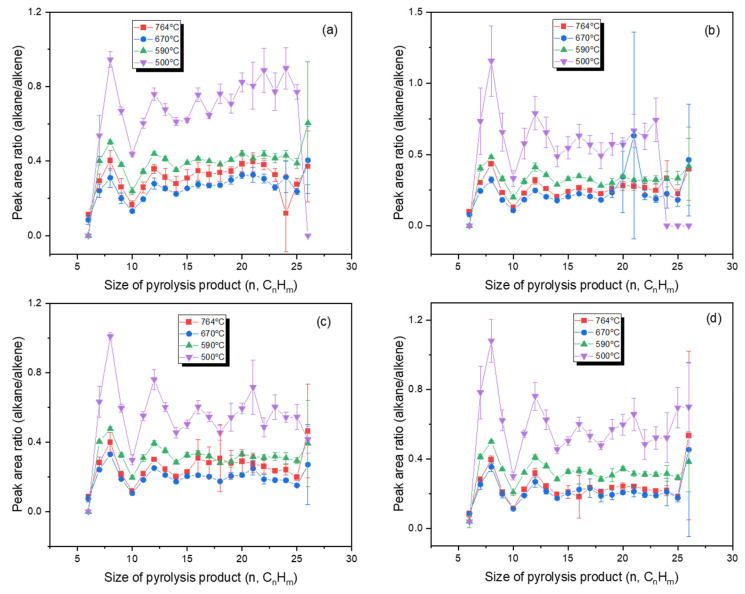
Variations in the peak intensity ratio of alkane/alkene with the size of pyrolysis product (carbon number) for PE2k (**a**), PE16k (**b**), PE28k (**c**), and PE56k (**d**).

The abundance ratios of alkane/alkene exhibited trends that are different from those of alkadiene/alkene (Figure 6). The alkane/alkene peak intensity ratios did not increase with the pyrolysis product size. The peak intensity ratios of alkane/alkene were lower than 1.0, except for the C8 species at 500 °C. All the alkane/alkene peak intensity ratios demonstrated a local maximum at C8 and a local minimum at C10. The order of the peak intensity ratio of alkane/alkene based on the pyrolysis temperature was 500 °C > 590 °C > 764 °C > 670 °C. The peak intensity ratios of alkane/alkene in the range of C11–C20 products did not change significantly. The peak intensity ratios of alkane/alkene for the C11–C20 products of the PE2k sample were 0.70 ± 0.12, 0.40 ± 0.05, 0.26 ± 0.06, and 0.37 ± 0.07 for the pyrolysis temperatures of 500 °C, 590 °C, 670 °C, and 764 °C, respectively. The peak intensity ratios for the C11–C20 products of the PE16k sample were 0.59 ± 0.20, 0.33 ± 0.08, 0.22 ± 0.12, and 0.25 ± 0.07; those of the PE28k sample were 0.56 ± 0.20, 0.32 ± 0.07, 0.20 ± 0.05, and 0.27 ± 0.07; and those of the PE56k sample were 0.57 ± 0.19, 0.33 ± 0.08, 0.21 ± 0.06, and 0.23 ± 0.09. The decreasing ratio observed with the increasing pyrolysis temperature indicates that the production of alkanes becomes less favorable at higher temperatures.

The formation of major pyrolysis products starts with the dissociation of the C-C bond in PE following the formation of CH_3_(CH_2_)_n_^•^ radicals. The energy-minimized structures of neutral species (C_30_H_62_, C_10_H_22_, C_10_H_20_, and C_10_H_18_), radicals (C_30_H_61_^•^ and CH_3_(CH_2_)_9_^•^), and a biradical (^•^(CH_2_)_10_^•^) were calculated based on the representative linear saturated hydrocarbon C_30_H_62_, and their energies were obtained to thermodynamically compare the formation of alkadiene, alkene, and alkane. Their energies are summarized in Table 1.

CH_3_(CH_2_)_9_^•^ + C_30_H_62_ → CH_3_(CH_2_)_8_CH_3_ + C_30_H_61_^•^  ΔH_rxn_ = −1 kJ/mol (1)

CH_3_(CH_2_)_9_^•^ → CH_3_(CH_2_)_7_CH=CH_2_ + H                         ΔH_rxn_ = 160 kJ/mol (2)

^•^(CH_2_)_10_^•^ → H_2_C=CH(CH_2_)_6_CH=CH_2_ + 2H           ΔH_rxn_ = 149 kJ/mol (3)

The heat of reaction for the formation of C_10_H_22_ from the CH_3_(CH_2_)_9_^•^ radical, which occurs by the abstraction of a hydrogen atom from the neutral C_30_H_62_ (reaction (1)), is −1 kJ/mol, which means that this reaction is thermodynamically favorable. However, the abundance of alkanes was lower than that of alkenes. This is because the formation of alkanes is the intermolecular reaction between an alkyl radical and a gas phase neutral PE. The probability of the alkyl radical to reacting with a PE molecule may not be high enough at any moment to generate an alkane. On the contrary, the formation of alkenes is an intramolecular reaction of rearranging following the loss of a hydrogen atom. The formation of an alkene from an alkyl radical (reaction (2)) is slightly less favorable than that of an alkadiene from a biradical (reaction (3)); however, the formation of a biradical from a neutral species requires twice the energy of a monoradical. Thus, the formation of an alkene is more favorable than that of an alkadiene.

**Figure 7:**
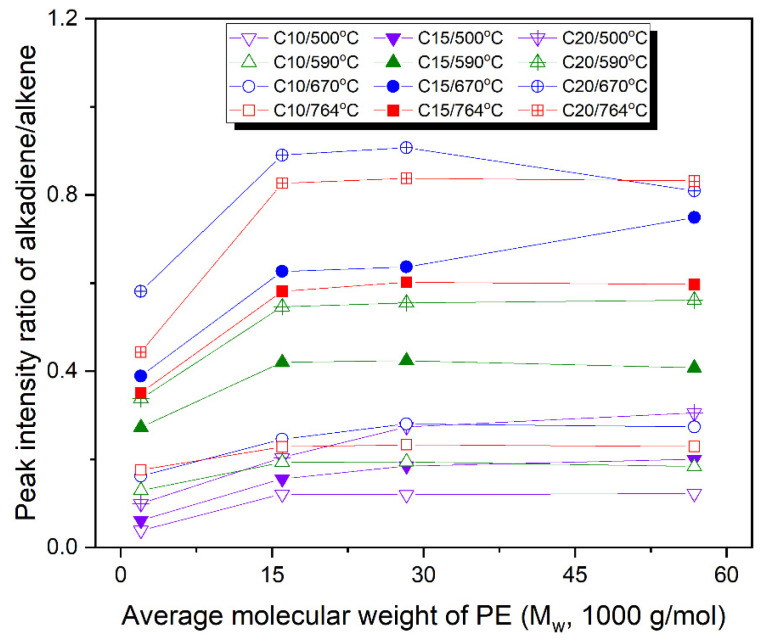
Variations in the peak intensity ratio of alkadiene/alkene of the C10, C15, and C20 products with PE molecular weight (M_w_). The open, solid, and cross symbols stand for the alkane/alkene ratios of the C10, C15, and C20 products, respectively. The squares, circles, upward-facing-triangles, and downward-facing-triangles denote the pyrolysis temperatures of 764 °C, 670 °C, 590 °C, and 500 °C, respectively.

**Figure 8:**
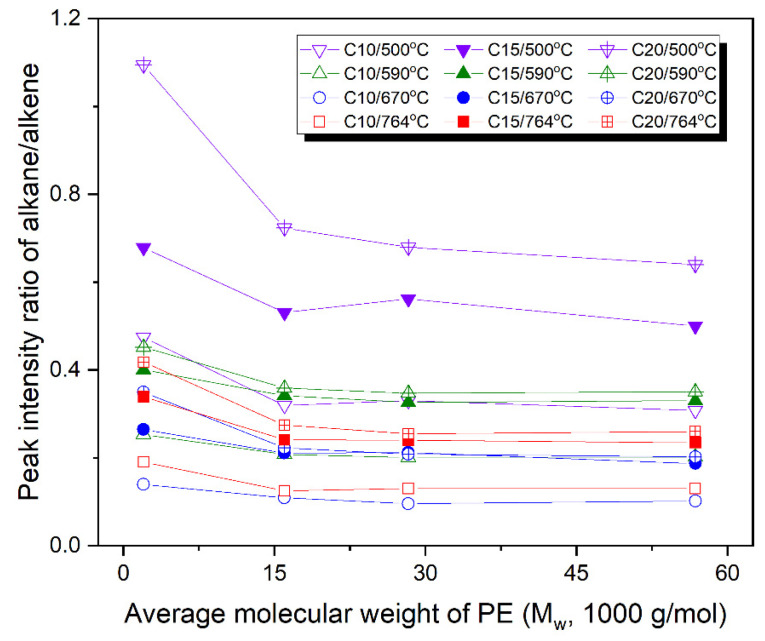
Variations in the peak intensity ratios of alkanes/alkenes of the C10, C15, and C20 products with PE molecular weight (M_w_). The open, solid, and cross symbols stand for the alkane/alkene ratios of the C10, C15, and C20 products, respectively. The squares, circles, upward-facing-triangles, and downward-facing-triangles denote the pyrolysis temperatures of 764 °C, 670 °C, 590 °C, and 500 °C, respectively.

To examine the influence of the PE molecular weight on the production rates of major pyrolysis products, the C10, C15, and C20 pyrolysis products were employed as representatives, and their variations in alkadiene/alkene and alkane/alkene ratios based on the PE molecular weight were plotted in Figure 7 and Figure 8, respectively. The alkadiene/alkene ratios were lower than 1.0, indicating that the formation of alkadienes is unfavorable compared to that of alkenes. The peak intensity ratios increased significantly as the M_w_ of PE increased from 2k to 16k Da, irrespective of the pyrolysis temperature and pyrolysis product size. After that, the changes in peak intensity ratios based on the M_w_ values were not significant, except in certain cases. At a low pyrolysis temperature of 500 °C, the alkadiene/alkene ratios continuously increased with M_w_, and the increasing trend became clearer as the pyrolysis product size increased. The alkadiene/alkene ratio increased with the size of pyrolysis products. This implies that the production of alkadienes becomes more favorable as their size increases, when compared to the production of alkenes.

Variations in the alkane/alkene ratios demonstrated opposite trends to those of the alkadiene/alkene ratios (Figure 8). The peak intensity ratios significantly decreased as the M_w_ value increased from 2k to 16k Da, while they tended to slightly decrease as the M_w_ value increased, except for the C15 product at 500 °C. These results lead to the conclusion that the formation of alkanes and alkenes from PEs with molecular weights higher than 20k Da is not significantly influenced by the molecular weight. The alkane/alkene ratios were lower than 1.0, except for the C20 case of the PE2k sample at 500 °C, which means that the formation of alkanes is less favorable than that of alkenes. The alkane/alkene ratios increased when the size of the pyrolysis products increased. This implies that the production of alkanes becomes more favorable as their size increases, when compared to the production of alkenes.

## 4. Conclusions

The major pyrolysis products of PE were alkadienes, alkenes, and alkanes, and the order of production was alkenes > alkanes > alkadienes. The abundance of alkadienes increased as their size increased from C_7_H_12_ to approximately C_20_H_38_, and then decreased. The pyrolysis temperature should be higher than 500 °C to produce alkadienes from PE. More alkenes and alkanes were produced at 590 °C than at other temperatures. The alkadiene/alkene ratio increased with the pyrolysis temperature until it reached 670 °C, indicating that the production of alkadiene became more favorable at high temperatures. Conversely, the alkane/alkene ratio decreased as the pyrolysis temperature increased until it reached 670 °C, indicating that the production of alkanes became less favorable at high temperatures. The alkadiene/alkene and alkane/alkene ratios were <1.0, indicating that the formation of alkadienes and alkanes was less favorable than that of alkenes. The alkadiene/alkene ratios tended to increase as the molecular weight of PE increased. The production of alkadienes became more favorable as their size increased. In contrast, the alkane/alkene ratios decreased as the molecular weight of PE increased. The production of alkanes became more favorable as their size increased.

## Figures and Tables

**Table 1:** Energies of the energy-minimized structures of neutral, radical, and biradical species based on C_30_H_62_ (kJ/mol).

Chemical	Energy (kJ/mol)
C_30_H_62_	−3,100,554
C_10_H_22_ (n-decane)	−1,035,633
C_10_H_20_ (1-decene)	−1,032,389
C_10_H_18_ (α,ω-decadiene)	−1,029,144
C_30_H_61_^•^	−3,098,790
CH_3_(CH_2_)_9_^•^	−1,033,868
^•^(CH_2_)_10_^•^	−1,031,931
H	−1319

## Data Availability

The data presented in this study are available upon request from the corresponding author. The data are not publicly available due to privacy reasons.
